# Venus-Earth-Mars: Comparative Climatology and the Search for Life in the Solar System

**DOI:** 10.3390/life2030255

**Published:** 2012-09-19

**Authors:** Roger D. Launius

**Affiliations:** Division of Space History, National Air and Space Museum; Smithsonian Institution, P.O. Box 37012, NASM Room 3550, MRC 311, Washington, DC 20013-7012, USA; E-Mail: launiusr@si.edu; Tel.: 202-633-2428

**Keywords:** Venus, Mars, Earth, space exploration, astrobiology, Percival Lowell, NASA, Carl Sagan, Percival Lowell, James C. Fletcher

## Abstract

Both Venus and Mars have captured the human imagination during the twentieth century as possible abodes of life. Venus had long enchanted humans—all the more so after astronomers realized it was shrouded in a mysterious cloak of clouds permanently hiding the surface from view. It was also the closest planet to Earth, with nearly the same size and surface gravity. These attributes brought myriad speculations about the nature of Venus, its climate, and the possibility of life existing there in some form. Mars also harbored interest as a place where life had or might still exist. Seasonal changes on Mars were interpreted as due to the possible spread and retreat of ice caps and lichen-like vegetation. A core element of this belief rested with the climatology of these two planets, as observed by astronomers, but these ideas were significantly altered, if not dashed during the space age. Missions to Venus and Mars revealed strikingly different worlds. The high temperatures and pressures found on Venus supported a “runaway greenhouse theory,” and Mars harbored an apparently lifeless landscape similar to the surface of the Moon. While hopes for Venus as an abode of life ended, the search for evidence of past life on Mars, possibly microbial, remains a central theme in space exploration. This survey explores the evolution of thinking about the climates of Venus and Mars as life-support systems, in comparison to Earth.

## 1. Introduction

This essay explores the evolution of thinking about the climates of Venus and Mars as life-support systems, in comparison to Earth. At some level this belief rests on little more than high expectations and exceptionally thin air, at least on Mars, as well as thick air on Venus [1]. It also suggests the central role of post-World War II scientific priorities, often dictated by national security concerns in the United States *vis à vis* the Soviet Union. The influence of Cold War institutions on postwar science, technology, and medicine was pervasive, and the search for life on Venus and Mars, while it had a legitimate scientific element, also engaged the attention of sponsors because of Cold War priorities. Those involved in the pursuit, as Audra Wolfe has shown, helped to define “the American space program as one with ‘scientifically valid’ goals” and at the same time demonstrated American capabilities [2].

## 2. Venerean Visions

Planetary exploration began in the early 1960s in a race between the United States and the Soviet Union to see who would be the first to place some sort of spacecraft near Venus. This was not just an opportunity to best the rival in the Cold War; scientists in both the United States and the Soviet Union recognized the attraction of Venus as a near twin to this planet in terms of size, mass, and gravitation. As Earth’s “sister planet,” a near twin, scientists and the public speculated about the nature of Venus and the possibility of life existing there in some form. Through much of the nineteenth century observers harbored hopes that Venus might be a place teeming with life. As R.A. Proctor wrote in 1870, because of its similarity to Earth “on the whole, the evidence we have points very strongly to Venus as the abode of living creatures not unlike the inhabitants of earth” [3,4].

In the latter part of the nineteenth century, however, a series of astronomical observations suggested Venus may be much less conducive to life similar to that seen on Earth than previously expected, in no small part because one side seemed to perpetually face the Sun, perhaps leaving one side too baked and the other too frozen for life [5,6]. Because of the thick clouds covering the planet’s surface, however, the observations were tentative and many scientists refused to accept this idea, in no small part because of the desire to believe that life lived at the bottom of that dense cloud cover. R.G. Aitken, an astronomer at Lick Observatory, argued that if Venus did rotate similar to the Earth “we have reason to believe that it is habitable, for the conditions we named as essential to life—air, water in its liquid form and a moderate temperature—are undoubtedly realized.” Absent that rotation, however, Aitken admitted that the possibility of life there “must be utterly desolate” [7]. 

Even so, and perhaps surprisingly, in the first half of the twentieth century a popular theory held that the sun had gradually been cooling for millennia and that as it did so, each planet in the solar system had a turn as a haven for life of various types. Although it was now Earth’s turn to harbor life, the theory suggested that Mars had once been habitable and that life on Venus was now just beginning to evolve. Beneath the clouds of the planet, the theory offered, was a warm, watery world and the possibility of aquatic and amphibious life. “It was reasoned that if the oceans of Venus still exist, then the Venusian clouds may be composed of water droplets,” noted JPL researchers as late as 1963; “if Venus were covered by water, it was suggested that it might be inhabited by Venusian equivalents of Earth’s Cambrian period of 500 million years ago, and the same steamy atmo­sphere could be a possibility” [8].

This theory was popularized by Svante Arrhenius, a Nobel Prize-winning chemist who reached millions with popular lectures and publications. Arguing for a tropical environment of more than 37.8 Celsius (100 degrees Fahrenheit) Arrhenius posited a strikingly wet atmosphere on Venus, one conducive to the rise of aquatic and amphibian life. He wrote:

We must therefore conclude that everything on Venus is dripping wet…A very great part of the surface of Venus is no doubt covered with swamps, corresponding to those on the Earth in which the coal deposits were formed…The constantly uniform climactic [sic] conditions which exist everywhere result in an entire absence of adaptation to changing exterior conditions. Only low forms of life are therefore represented, mostly no doubt belonging to the vegetable kingdom; and the organisms are nearly of the same kind all over the planet. The vegetative processes are greatly accelerated by the high temperatures.

Arrhenius speculated that more complex life forms might have evolved at the Venusian poles since the temperatures would not be quite as hot there, and with that “progress and culture...will gradually spread from the poles toward the equator” [9].

The director of Smithsonian observatory, Charles Greeley Abbot, took these ideas even further. He argued concerning Venus, a twin of Earth, in the 1920s that its “high reflecting power seems to show that Venus is largely covered by clouds indicative of abundant moisture, probably at almost identical temperatures to ours.” He then concluded that it “appears lacking in no essential to habitability” [10]. For Abbot, Venus was even more attractive an abode of life than Mars and he made the case in several studies that followed. He even speculated that Earth might make contact with Venereans, evincing his excitement at coming “into fluent communication by wireless with a race brought up completely separate, having their own systems of government, social usages, religions, and surrounded by vegetation and animals entirely unrelated to any here on earth. It would be a revelation far beyond the opening of Japan, or the discoveries of Egyptologists, or the adventures of travelers in the dark continent” [11]. Of course, spectroscopic investigation, and the failure by this means to find oxygen in the Venerean cloud system, made many scientists question Venus as an abode of life. 

Thus the debate over the climate of Venus portended a larger debate over the possibilities of life in the solar system. Venus’s atmosphere, the pressures it had, the presence or absence of atmospheric oxygen, H2O, and CO2 fundamentally informed this debate. It led to a succession of planetary theories concerning Venus. Measurement of these climate characteristics constrained theoretical models of planetary evolution while also restraining some of the more exotic speculations about Martian and Venerean life.

By the 1930s the detection of carbon dioxide in its thick atmosphere forced scientists grudgingly to abandon the idea that Venus contained a carboniferous swamp. The scientists investigating Venus replaced the pre-Cambrian environment, as Carl Sagan noted in 1961, for “an arid planetary desert, overlain by clouds of dust from the wind-swept surface” [12]. They continued to search for water vapor, but failed to find it. What scientists found was carbon dioxide, a lot of it; a layer of gas roughly equivalent to a two mile deep ocean at a pressure similar to that of Earth [13,14]. In 1939 astronomer Rupert Wildt postulated a “greenhouse effect” with temperatures far above what was present on Earth. As astronomer Ronald A. Schorn concluded, “By 1940 there was good reason to believe that conditions on Venus were harsh and life impossible” [15]. Charles Greeley Abbot, for one, refused to change his perspective. He offered concerning Venus as late as 1946 that “the conditions may possibly be as favorable for life there as on our earth” [16].

Few scientists wanted to accept that conclusion, however, at least as yet. Astronomer/cosmographer/iconoclast Fred Hoyle tried to explain away the lack of water. According to Carl Sagan:

Hoyle explained the lack of water by assuming a great excess of hydrocarbons over water on primitive Venus, and subsequent oxidation of the hydrocarbons to carbon dioxide, until all the water was depleted. He suggested that the surface is now covered with the remainder of the hydrocarbons, and that the cloud layer is composed of smog [17].

In such an environment, the planet Venus might be a global petroleum field awaiting human exploitation. Some advocates even suggested mining it for energy.

Even so, as late as the middle part of the 1950s Donald H. Menzel and Fred L. Whipple found another explanation for the data that allowed the possibility, however narrow, that life might have emerged on Venus by replacing both the wind-swept desert and planetary oil field theories with speculation that Venus was covered with a seltzer ocean. By arguing for a global seltzer ocean, Menzel and Whipple suggested that the chemical processes of the transformation of CO2 into silicates would be hampered. Stranger things had happened, but many other scientists refused to accept this theory, and Otto Struve even called it unworthy of serious discussion [18,19].

In fits of wishful thinking, a few others continued to claim that they saw signs of life through their telescopes. Soviet astronomer G.A. Tikhoff in 1955 emphasized:

Now already we can say a few things about the vegetation of Venus. Owing to the high temperatures on this planet, the plants must reflect all the heat rays, of which those visible to the eye are the rays from red to green inclusive. This gives the plants a yellow hue. In addition, the plants must radiate red rays. With the yellow, this gives them an orange color. Our conclusions concerning the color of vegetation on Venus find certain confirmation in the observation…that in those pats of Venus where the Sun’s rays possibly penetrate the clouds to be reflected by the planet’s surface, there is a surplus of yellow and red rays [20].

Subsequent measurements largely subverted the idea of Venus as a planet teeming with life even before the dawn of interplanetary travel.

**Figure 1:**
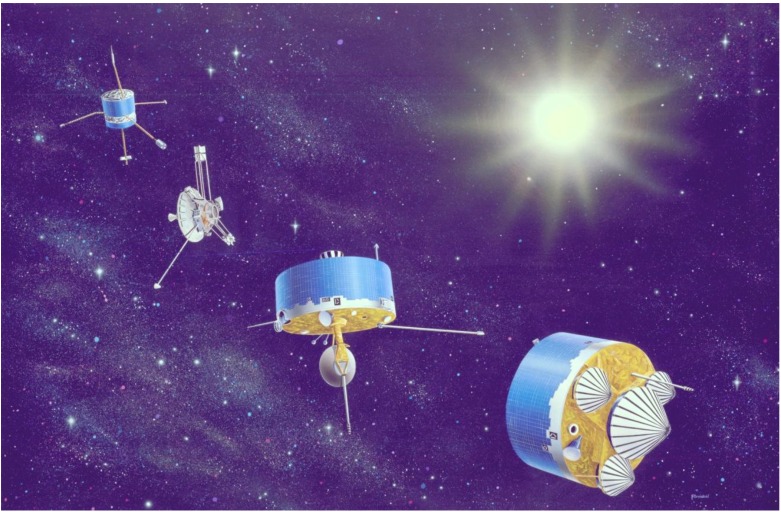
Pioneer Group Portrait.

So bleak did the situation appear by 1961, at the point when the first spacecraft were being dispatched to Venus, Figure 1, that even Carl Sagan thought it unlikely that the planet has ever harbored life. As he concluded:

At such high temperatures, and in the absence of liquid water, it appears very unlikely that there are indigenous surface organisms at the present time. If life based upon carbon-hydrogen-oxygen-nitrogen chemistry ever developed in the early history of Venus, it must subsequently have evolved to sub-surface or atmospheric ecological niches. However, since, as has been mentioned, there can have been no appreciable periods of time when Venus had both extensive bodies of water and surface temperatures below the boiling point of water, it is unlikely that life ever arose on Venus [21].

Sagan’s conclusion squared with a Space Science Board’s analysis completed near the same time [22].

A family portrait showing (from left to right) Pioneers 6–9, 10 and 11 and the Pioneer Venus Orbiter and Multiprobe series. These were the stalwarts of the missions to Venus and Mars until the 1990s. (Credit: NASA)

This would have been a difficult conclusion for Sagan to accept judging from a career dedicated to finding life beyond Earth, and even here he held out for the prospect of reengineering the planet for biological habitation. He concludes:

Ideally, we can envisage the seeding of the upper Cytherean atmosphere with appropriate strains of Nostocaceae [a family of cyanobacteria that forms filament-shaped colonies enclosed in mucus or a gelatinous sheath] after exhaustive studies have been performed on the existing environment of Venus. As the carbon dioxide content of the atmosphere fails, the greenhouse effect is rendered less efficient and the surface temperature fails. After the atmospheric temperatures decline sufficiently, the decreasing rate of algal decomposition will reduce the water abundance slightly and permit the surface to cool below the boiling point of water….At somewhat lower temperatures, rain will reach the surface, and the Urey equilibrium will be initiated, further reducing the atmospheric content of carbon dioxide to terrestrial values. With a few centimeters of perceptible water in the air, surface temperatures somewhere near room temperature, a breathable atmosphere, and terrestrial microfiora awaiting the next ecological succession, Venus will have become a much less forbidding environment than it appears to be at present [23].

Such a faith statement in the face of the wealth of countervailing evidence is breathtaking for scientists dedicated to the analysis of objective data.

After carrying out ground-based efforts in 1961 to view the planet using radar, which could “see” through the clouds, and learning among other things that Venus rotated in a retrograde motion opposite from the direction of orbital motion, both the Soviet Union and the United States began a race to the planet with robotic spacecraft to learn the truth about the planet and its prospects for life. The Soviets tried first, launching Venera 1 on February 12, 1961. Unlike lunar exploration, however, the Soviets did not win the race to Venus; their spacecraft broke down on the way. The United States claimed the first success in planetary exploration during the summer of 1962 when Mariner 1 and Mariner 2 were launched toward Venus. Although Mariner 1 was lost during a launch failure, Mariner 2 flew by Venus on December 14, 1962, at a distance of 30,000 km (21,641 miles). 

It investigated the clouds, estimated planetary temperatures, measured the charged particle environment, and looked for a magnetic field similar to Earth’s magnetosphere (but found none). Most important, it confirmed that the planet’s surface was an inferno:

Earth-based measurements of microwave emissions from Venus had indicated a temperature of about 600 °F., but researchers did not—and could not—know whether the emissions came from the surface, from cloud layers in the atmosphere or from a dense ionosphere high overhead. The question was answered by a microwave radiometer aboard Mariner 2, which revealed “limb-darkening” (weaker emissions at the edge of the planet’s disk than at the center). The conclusion was not only that the surface was the hot part, but that, at about 800 °F., it was even hotter than the earth-based data had implied. An infrared radiometer, meanwhile, took temperatures high in the atmosphere, revealing, to the scientists’ disappointment, no breaks in the clouds [24].

Certainly, such an environment made unlikely the theory that ­life—at least as humans understood ­it—had ever existed on Venus.

Although the Soviet Union made several attempts to reach Venus, only in 1965 was it successful in reaching the surface when Venera 3 crashed there without returning any scientific data. In 1967 the Soviets sent Venera 4, which successfully deployed a probe into the atmo­sphere and returned further information about the makeup of the planet’s surface. In the same year the Americans sent Mariner 5 to Venus to investigate its atmosphere. Both spacecraft demonstrated that Venus was a very inhospitable place for life to exist. Through the late 1960s, the Soviets continued to send probes to Venus to obtain data from the inhospitable surface, but none succeeded until Venera 7 returned the first information from the surface in December 1970. For 23 minutes, the lander returned data and imagery about conditions on the ground before succumbing to the extreme heat and pressure. It was the first time that any lander had returned information from the surface of another planet [25].

Collectively, these and subsequent planetary spacecraft revealed that Venus was superheated because of the greenhouse effect of the cloud layer, and that the pressure on the surface was about 90 atmospheres, far greater than even in the depths of the oceans on Earth. Add to this the observations of James Pollack and others using aircraft-based near-infrared spectroscopy in 1974 that found on Venus a cloud sheet made predominantly of sulfuric acid, and the possibilities of life on the planet appeared as remote as they had ever been [26].

Even with what was already known about Venus, the planet remained a priority for exploration, and because of Venus’s thick cloud cover, scientists early on advocated sending a spacecraft with radar to map Venus. Pioneer 12 had made a start toward realizing that goal, orbiting the planet for more than a decade to complete a low-resolution radar topographic map. Likewise, the Soviets’ Venera 15 and 16 missions in 1983 provided high-resolution coverage over the northern reaches of the planet. The best opportunity to learn the features of the Venerean surface came in the early 1990s with what turned out to be a highly successful Magellan mission to Venus. Launched in 1989, Magellan mapped 95 percent of the surface at high resolution, parts of it in stereo. This data provided some surprises; among them the discoveries that plate tectonics was at work on Venus and that lava flows showed clear evidence of volcanic activity. For five years Magellan yielded outstanding scientific results, showing volcanoes, faults, impact craters, and lava flows. It failed to deliver any data that suggested possibilities for life on the planet [27]. 

Although one would think that evidence from the spacecraft sent to Venus would be conclusive, overwhelmingly altering most of the beliefs held as recently as a generation ago about Venus as an abode of life, such was only partially true. For example, data from the Pioneer Venus mission suggested that in the distant past Venus had an ocean that may have existed for as long as a billion years, certainly enough to spawn primitive life, before sublimating the moisture into space. Planetary scientist Thomas M. Donahue, University of Michigan in Ann Arbor, reported that he and his team of researchers had found traces of water molecules in the upper atmosphere of Venus. As *Science News* reported in 1993:

This chemical signature comes from the abundance of two atoms—hydrogen and its less abundant isotope deuterium, which has twice hydrogen’s mass.…The craft’s early measurements revealed that this deuterium-to-hydrogen ratio is at least 150 times greater on Venus than in any other known place in the solar system….And since hydrogen readily bonds with oxygen to produce water, this suggests that Venus once had a minimum of 150 times as much water as it does now.

Donahue concluded, “The data indicate that Venus was a pretty wet planet.” If this is proven correct, it may signal another shift in thought about Venus as a place where life might once have resided, even if conditions are no longer conducive to its survival [28].

With the same evidence from Venus being interpreted in different ways, the scientific community has long divided into two large groups when considering prospects for life on Earth’s “twin.” Essentially, some insisted that large amounts of water vapor existed on Venus, as much as 100 microns of perceptible vapor, while others insisted that results showed a lack of water. There has been no resolution of the debate, although the preponderance of evidence is in favor of a lifeless Venus. But some hope remains, because the community fundamentally wants to hope, and the 1993 announcement about water vapor in the Venerean atmosphere is one shred that enabled some hopefuls to cling to this belief despite the plethora of countervailing evidence. For most, however, beliefs held about Venus as a tropical, proto-organic planet had proven a bust.

## 3. The Lure of the Red Planet

But what of Mars? No planet has been more consistently held up as a possible location for life than the red planet. As with Venus, there had long been speculation of intelligent life there, and astronomical observations had lent credence to the idea, at least in the public mind. For instance, Percival Lowell became interested in Mars during the latter part of the nineteenth century. Using personal funds and grants from other sources, he built what became the Lowell Observatory near Flagstaff, Arizona, to study the planets. His research led him to argue that Mars had once been a watery planet and that the topographical features known as canals had been built by intelligent beings. Over the course of the first forty years of the twentieth century, others used Lowell’s observations of Mars as a foundation for their arguments. The idea of intelligent life on Mars stayed in the popular imagination for a long time, and it was only with the scientific data returned from robots sent to the planet since the beginning of the space age that this began to change [29].

The United States had reached Mars by July 15, 1965, when Mariner 4 flew within 10,000 km (6,118 mi) of the planet taking twenty-one close-up pictures. These photographs dashed the hopes of many that life might be present on Mars, for these first close‑up images of Mars showed a cratered, lunar‑like surface. They depicted a Mars without artificial structures and canals, nothing that even remotely resembled a pattern that intelligent life might produce. *U.S. News and World Report* announced that “Mars is dead” [30]. Even President Lyndon Johnson pronounced that “life as we know it with its humanity is more unique than many have thought” [31]. Since the vast distance to the nearest star precluded visits to another solar system in the foreseeable future, life scientists gave up their hope of direct contact with other intelligent life on Mars and focused on the dual search for evidence that it had once existed there and that non-intelligent or microbial life existed there [32].

Mariners 6 and 7, launched in February and March 1969, each passed Mars in August 1969, studying its atmosphere and surface to lay the groundwork for an eventual landing on the planet. Their pictures verified the Moon‑like appearance of Mars, but they also found that volcanoes had once been active on the planet, that the frost observed seasonally on the poles was made of carbon dioxide, and that huge plates indicated considerable tectonic activity in the planet’s past. Mariner 9 entered Martian orbit in November 1971, and its pictures showed the remains of giant extinct volcanoes dwarfing anything on the Earth. Later pictures showed a canyon, “Valles Marineris,” 4,000 km (2,500 mi) long and 6 km (3.5 mi) deep, and meandering “rivers,” indicating that, at some time in the past, fluid had flowed on Mars. Suddenly, Mars fascinated scientists, reporters, and the public once again, largely because of the possibility of past life that might have emerged because of the evidence of flowing water [33].

To find the answer about the Martian past, and perhaps its present as well, NASA developed what became Project Viking, a soft landing mission to Mars, but it also included what turned out to be two significant Mars orbiters that mapped the surface. Very clearly, the search for signs of life prompted this emphasis on the exploration of Mars. NASA Administrator James C. Fletcher, for example, supported the Viking mission because of his belief that life was present in the universe and that greater knowledge of this probably might be found on Mars:

Although the discoveries we shall make on our neighboring worlds will revolutionize our knowledge of the Universe, and probably transform human society, it is unlikely that we will find intelligent life on the other planets of our Sun. Yet, it is likely we would find it among the stars of the galaxy, and that is reason enough to initiate the quest....We should begin to listen to other civilizations in the galaxy. It must be full of voices, calling from star to star in a myriad of tongues. Though we are separate from this cosmic conversation by light years, we can certainly listen ten million times further than we can travel [34].

Fletcher supported the Viking Mars lander in part because of its biological research on the red planet.

The Viking mission consisted of two identical spacecraft, each consisting of a lander and an orbiter. Launched in 1975 from the Kennedy Space Center, Florida, Viking 1 spent nearly a year cruising to Mars, placed an orbiter in operation around the planet, and landed on July 20, 1976, on the Chryse Planitia (Golden Plains), with Viking 2 following in September 1976. While one of the most important scientific activities of this project involved an attempt to determine whether there was life on Mars, the scientific data returned mitigated against the possibility. The two landers continuously monitored weather at the landing sites and found both exciting cyclical variations and an exceptionally harsh climate that mitigated the possibility of life. Atmospheric temperatures at the more southern Viking 1 landing site, for instance, were only as high as +13.9 Celsius (+7 degrees Fahrenheit) at midday, but the predawn summer temperature was −17.8 Celsius (−107 degrees Fahrenheit). And the lowest predawn temperature was −184 degrees Fahrenheit, about the frost point of carbon dioxide [35]. 

Although the three biology experiments discovered unexpected and enigmatic chemical activity in the Martian soil, they provided no clear evidence for the presence of living microorganisms in soil near the landing sites. According to mission biologists, Mars was self-sterilizing. They concluded that the combination of solar ultraviolet radiation that saturates the surface, the extreme dryness of the soil, and the oxidizing nature of the soil chemistry had prevented the formation of living organisms in the Martian soil [36]. The uncertainty of the conclusions from Viking haunted the program’s chief scientist, Gerald Soffen, ever after. He was known to second guess his judgment; perhaps he should have installed a microscope on the lander. But, he also believed he did the best he could. “I think what we did was ahead of our time. We were young enough not to know that it couldn’t be done,” Soffen recalled [37].

Viking found no evidence of surface life, or even life that might live at the depths that the lander could dig on the Martian surface, but as it turns out, that should not have been surprising. Surface dwellers are probably rare. On Earth, most of the biomass lives below the planetary surface in the soil or the oceans. Regardless of negative results from the Viking landers, this fact offered something for scientists to cling to as they considered future exploration of the red planet.

The failure to find evidence of life on these two planets in the Solar System devastated the optimism present for astrobiology in the era of great expectations. Collectively, these missions led to the development of two essential reactions. The first was a questioning by a significant minority of scientists that complex life might not exist elsewhere in the Solar System, but that did not mean that it was not present throughout the universe. While scientists grew discouraged, it was a disappointment that did not remain for many of them. JPL director Bruce Murray believed that the legacy of failure to detect life, despite the billions spent and a succession of overoptimistic statements, would spark public disappointment and perhaps a public outrage [38].

**Figure 2:**
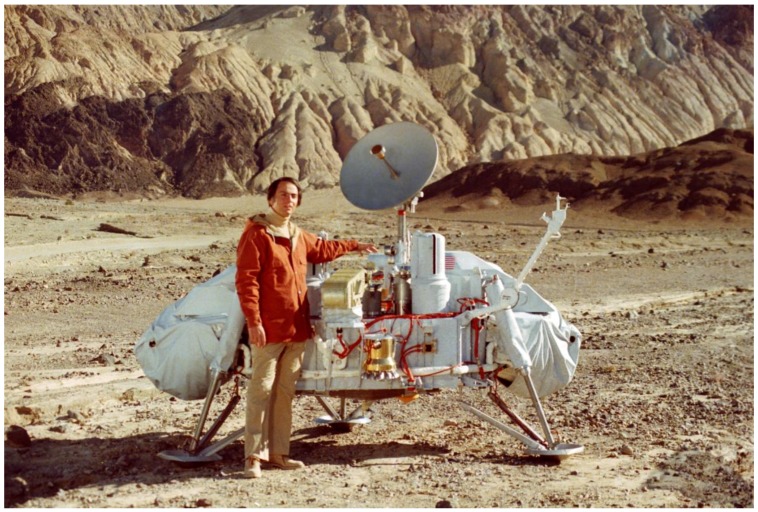
Carl Sagan with the Viking lander mock-up in Death Valley, California, on October 26, 1980. (Credit: Jet Propulsion Laboratory/NASA)

At least by the Viking landings in 1976 it began to be seen that the prospects for discovering extraterrestrial life on Mars had been oversold. Planetary scientist and JPL director Bruce Murray complained at the time of Viking about the lander being ballyhooed as a definite means of ascertaining whether or not life existed on Mars. The public expected to find it, and probably so did many of the scientists, and what would happen when hopes were dashed? Murray argued that “the extraordinarily hostile environment revealed by the Mariner flybys made life there so unlikely that public expectations should not be raised.” Carl Sagan, Figure 2, who fully expected to find something there, accused Murray of pessimism. Murray accused Sagan of far too much optimism. And the two publicly jousted over how to treat the Viking mission. Murray, as well as other politically savvy scientists and public intellectuals, argued that the legacy of failure to detect life, despite billions spent on research since the beginning of the space age and overoptimistic statements that a breakthrough was just around the corner, would spark public disappointment and perhaps an outrage manifested in reduced public funding for the effort [39].

The failure of Viking to find evidence of life on Mars revealed a core problem of overselling possibilities for extraterrestrial life and its discovery. The disappointment was palpable, at least if missions are sparked by success. Thereafter, no spacecraft went to Mars for more than twenty years after Viking. Not until 1988 did the Soviet Union, just a year away from collapse and the end of the Cold War, send Phobos 1 and 2 to Mars, but both failed enroute to Mars. The Mars Observer launched by the United States on September 25, 1992, fared little better. Intended to provide the most detailed data available about Mars as it orbited the planet since what had been collected by the Viking explorers of the mid-1970s, the mission was progressing smoothly until August 21, 1993, three days before the spacecraft’s capture in orbit around Mars. Suddenly, and without warning, controllers lost contact with it. The engineering team working on the project at the Jet Propulsion Laboratory responded with a series of commands to turn on the spacecraft’s transmitter and to point the spacecraft’s antennas toward Earth. No signal came from the spacecraft, however, and the Mars Observer was not heard from again. The loss of the nearly $1 billion Mars Observer probably came as a result of an explosion in the fuel lines of the space vehicle. One wit offered an alternative explanation, suggesting that after the landing by the Vikings in 1976 the Martians had developed a planetary defense system and it was now knocking out everything aimed at the red planet. 

Even though all of the elements of disillusionment remained present in the mid-1990s, those who had created heightened expectations seem to have learned from their errors, and conspiracy theorists seem to have been increasingly discredited in popular conception. But most important, activities in several related fields of astrobiology seem to be offering renewed hope for discovering extraterrestrial life. Earthly studies have helped to show additional possibilities for extraterrestrial life, as marine biologists have discovered life—even though very far removed from what we have known before—in the depths of the ocean, under polar caps, and in strata previously thought uninhabitable. Might creatures of such type be able to survive in what we have usually thought of as barren planets such as Mars and Venus? 

A change to the beliefs in life on Mars took place in August 1996 when a team of NASA and Stanford University scientists announced that a Mars meteorite found in Antarctica contained possible evidence of ancient Martian life. When the 2 kilogram (4.2 pounds), potato-sized rock, identified as ALH84001, formed as an igneous rock about 4.5 billion years ago, Mars was much warmer and probably contained oceans hospitable to life. Then, about 15 million years ago, a large asteroid hit the Red Planet and jettisoned the rock into space where it remained until it crashed into Antarctica about 11,000 B.C. The scientists presented three intriguing, but not conclusive, pieces of evidence that suggest that fossil-like remains of Martian microorganisms, which date back 3.6 billion years, are present in ALH84001. While there is no consensus on the truth of these findings, they did lead to added support for an aggressive set of missions to Mars to help discover the truth. Even as scientists modestly called for more research, the findings electrified the public and set in motion popular support for an aggressive set of missions to Mars by the year 2010 to help discover the truth of these theories [40].

Thereafter the strategy for much of Mars exploration has been built upon the motto, “Follow the Water.” In essence, this approach noted that life on Earth is built upon liquid water and that any life elsewhere would probably have chemistries built upon these same elements. Accordingly, to search for life on Mars, past or present, NASA’s strategy would be to follow the water. If scientists could find any liquid water on Mars, probably only deep beneath the surface, the potential for life to exist was also present [41]. 

The Mars of today, without any evidence of water whatsoever on the surface, probably had water flowing freely in its ancient history. One scientist concluded as early as 1988: “Even though the Martian epoch of liquid water was short, it apparently coincided with the period of Earth history when life originated. Gross similarities in the early geophysical history of the two planets hold open the possibility that life arose on Mars as well.… (A discussion of the biological potential of Venus, whose early geophysical history bears resemblance to that of Mars, would lead to the same conclusion.) Therefore, the search for evidence of extinct life on Mars should be among the highest scientific priorities in future explorations of the planet” [42].

Evidence of changes to the planet’s surface from fast flowing water has been collected by many satellites orbiting the planet since the latter 1990s. The spacecraft to open this possibility was Mars Global Surveyor, reaching the planet in 1998 and a new and exciting era of scientific missions to study the red planet. Its recent discoveries offer titillating hints for learning about the possibility of life on Mars, at least in the distant past. In an exciting press conference in June 2000, astronomer Michael Malin discussed his analysis of imagery from Mars Global Surveyor, a stunningly successful NASA mission. He showed more than 150 geographic features all over Mars probably created by fast flowing water. He suggested that there might actually be water in the substrata of Mars, and our experience on Earth has indicated that where water exists, life as we understand it exists as well [43]. 

Operating for several years, Mars Global Surveyor continued to send back views of the Martian surface that seemed to show evidence of dry riverbeds, flood plains, gullies on Martian cliffs and crater walls, and sedimentary deposits that suggested the presence of water flowing on the surface at some point in the history of Mars. This led scientists to theorize that billions of years ago, Earth and Mars might have been very similar places. Of course, Mars lost its water and the question of why that might have been the case has also motivated many Mars missions to the present. At that point, a consensus emerged that on any mission to Mars we should “follow the water” and seek the answer to the ultimate question: “Are we alone in the universe?” 

At present, most scientists believe the odds are almost nonexistent that complex life forms could have evolved on Mars because of its extremely hostile environment. The stories of “advanced civilizations,” as proposed by Percival Lowell, or “little green men” are just that, stories. But many scientists believe there is sufficient evidence to think that microscopic organisms might once have evolved on the planet when it was much warmer and wetter billions of years ago. There are even a few scientists who would go somewhat further and theorize that perhaps some water is still present deep inside the planet. In that case simple life forms might still be living beneath Mars’ polar caps or in subterranean hot springs warmed by vents from the Martian core. These might be Martian equivalents of single-celled microbes that dwell in Earth’s bedrock. Scientists are quick to add, however, that these are unproven theories for which evidence has not yet been discovered. This strategy of “follow the water” has dominated all planning for Mars science missions for more than a decade and results thus far have encouraged many to believe that the discovery of incontrovertible evidence supports the contention that life will be found on Mars, it is just a matter of time [44]. 

## 4. The Desire to Believe and the Continuing Search for Life on Venus and Mars

When the twentieth century began, the idea of a solar system brimming with life had a foothold in the collective consciousness of the American people. Moreover, many in the scientific community accepted the “fact” of life on Venus and Mars. While a completely unproven assertion, and unprovable using the scientific tools available at the time, the belief persisted. It also enjoyed a rather variegated reputation as a province of cranks, philosophers, and almost anyone other than scientists. At the end of the twentieth century, what has changed? The belief in extraterrestrial life is more firmly ensconced in the collective consciousness of the public and has gained legitimacy in the scientific world. There is still no proof acceptable to all, but there have been signs of possibility—notably the evidence from Mars—and the tools, methods, and scientific wherewithal to determine with some accuracy exists. In the more than forty years since the beginning of the space age, the best attempts of science to answer this age-old question have yielded contested results in the Solar System, enticing possibilities for life among the stars and the emergence of a cosmology that gives hope for a life-filled universe.

In the First Annual Carl Sagan Memorial Lecture in December 1997 before the American Astronautical Society, Bruce Murray summarized many of the possibilities for the future of “The Search for Life Elsewhere.” He described that search as a “unifying idea” of what space science has before it, and he was guardedly optimistic. He offered a fivefold program for the future:

(1) Complete the search for life in our Solar System, especially beneath the surface of Mars and on Jupiter’s moon Europa;
(2) Complete the search for preserved evidence of past life on the surface of Mars;
(3) Inventory planetary bodies orbiting other stars;
(4) Identify Earth-like planets around those stars, and search for spectral indications of life in their atmospheres; and
(5) Expand SETI to Earth orbit for submillimeter and shorter wavelength searches.

Humanity has come far in understanding about this issue since the beginning of the space age, Murray declared, and should be ready for exciting new efforts in the future [45].

During this forty year effort, the separate disciplines of Solar System Astrobiology, Origins and Evolution of Life, Planetary Systems, and SETI have merged into a powerful force for scientific research and study. Equally important, the necessary ingredients for any exploration—political will, economic stability and growth, enabling technologies, scientific sophistication necessary to conceptualize and incorporate new findings into the scientific paradigm, and public perception that these efforts are both achievable and desirable—have coalesced. At the beginning of the twenty-first century a modest, optimistic, and rational approach seems to offer a bright future for a discipline and for the discovery of extraterrestrial life, perhaps even on Mars. 

But one must ask why that may be the case. With a succession of inconclusive results at every point that spacecraft reached other worlds, especially Venus and Mars, why the persistence in the belief of life beyond Earth? As a truly unusual aspect of the studies of Venus and Mars, despite no clear evidence to support this belief after more than fifty years of scrutiny, a perception that life probably exists in the solar system at some point in the past, if not presently, remains a powerful ideal among many planetary scientists. In essence, they have followed a classic cognitive dissonance model, defined by Leon Festinger in his seminal 1956 book, *When Prophecy Fails*. Festinger asked the question, ‘What happens when a prediction to which a social group subscribes fails completely and without ambiguity? What happens to all its faithful supporters?’ Reason would suggest that the members of the group would abandon the commitments that proved faulty, adjusting their perspectives to reflect mainstream ideals. But true believers do not automatically abandon their cause when reality intrudes in discomforting ways. They rarely admit that they were wrong or change their behavior, especially those who remain close to the original group. As Festinger found, groups holding firm beliefs, however disconfirmed they might be by fact, were nonetheless committed at considerable expense to maintain it. He wrote, “If more and more people can be persuaded that the system of belief is correct, then clearly it must after all be correct.” Instead they increase their level of proselytizing, working hard to spread the underlying belief. They seek out new evidence to validate their old behavior and new explanations as to why initial expectations failed. Sometimes they even deny that their prophecies in fact did fail [46].

Festinger’s model works well with the scientific community using comparative climatology to postulate life on Venus and Mars. Most of them have made little attempt to deny their failure, but they rationalized what had taken place and restructured their perspectives without abandoning them. They did not give up their faith in life on Mars and Venus; rather, they changed their ideas on what it would consist of and the structure it would take. This is a fascinating development. 

The case of Mars is the most straightforward. Numerous distinguished scientists argued for civilizations on Mars during the pre-space age, in no small part because of the fabled canals advocated by Percival Lowell and others. As scientific instruments became more capable, they proved that those canals were nonexistent, but scientists did not abandon their belief in life on the planet, suggesting as late as the 1950s that vegetation changed colors through planetary seasons on Mars. Gerard P. Kuiper, using a powerful ground-based telescope at the McDonald Observatory, claimed to see a “touch of moss green” on Mars, sending fellow astronomers searching for a chlorophyll signature using spectroscopic analysis [47]. Some claimed to find it and for several years scientists reported that vegetation on the plant, probably lichens or some other type of plant life, changed with the seasons. These vivid colors finally proved illusory, and the greens and blues reported turned out to be visual tricks when neutral-toned areas were surrounded by yellow-orange dust storms [48]. 

When missions such as Mariner 4 reached Mars in the mid-1960s and revealed a cratered, Moon-like planet, scientists had to dial back their expectations yet again. But many did not abandon their beliefs. What about the potential for microbial life? The Viking landing mission was predicated on the belief that microbial life would be found in the Martian surface. Failure to discover any microbes proved devastating to the cause of life on Mars, but still some clung to the hope that they had looked in the wrong locations, or had designed the experiments in a way that did not yield useful results. But the idea did not completely die. By the 1990s, scientists had begun to approach the issue of life on Mars in another way, modifying but not abandoning earlier conceptions of a solar system containing life beyond Earth. They admitted that liquid water on the surface of Mars would either freeze or evaporate almost immediately, and that the atmosphere was also almost waterless. Even so, they asserted that features seen from space looked like they had been carved by rivers and fast-flowing floods. The last decade of the twentieth century brought new possibilities as data from the ALH84001 meteorite and the mission of the Mars Pathfinder spacecraft showed the potential of past liquid water flowing freely on the planet. With water as the fundamental building block of life, the search for life on Mars entered a new arena, a scaling back to past life now extinct on a dead world. Scientists may yet find it; certainly the evidence of past water on the planet is compelling. If fossils of prehistoric Martian creatures are found, it will hold important—even profound—implications for humanity. But the important point is that with every disconfirming piece of evidence about the lack of life on the planet, many scientists do not abandon hope; instead they modify the desire just enough to continue their hope for life beyond Earth. 

Recent disconfirming evidence of life elsewhere led scientists to modify their expectations yet again, asking the simple question—so simple that one must ponder why it has not been asked before—about whether or not humanity would recognize extraterrestrial life were they to encounter it. As a press release announcing a 2007 National Academies study noted concerning the search for life in the solar system:

The tacit assumption that alien life would utilize the same biochemical architecture as life on Earth does means that scientists have artificially limited the scope of their thinking as to where extraterrestrial life might be found, the report says. The assumption that life requires water, for example, has limited thinking about likely habitats on Mars to those places where liquid water is thought to be present or have once flowed, such as the deep subsurface. However, according to the committee, liquids such as ammonia or formamide could also work as biosolvents—liquids that dissolve substances within an organism—albeit through a different biochemistry [49].

John Baross, professor of oceanography at the University of Washington, commented on this shift: “The search so far has focused on Earth-like life because that’s all we know, but life that may have originated elsewhere could be unrecognizable compared with life here. Advances throughout the last decade in biology and biochemistry show that the basic requirements for life might not be as concrete as we thought.” Broadening the search might yield positive results, according to this report [50].

Interestingly, the possibility that life might not be out there never really entered into the discussion. Mars and Venus revealed themselves to be far more forbidding places than anticipated when the space age began. The Earth, by comparison, appears to be a far more unique and precious sphere. Few want to embrace this as a possibility, although a few scientists do. In their book *Rare Earth*, Peter Ward and Donald Brownlee suggest that complex life forms like those found on Earth require local conditions that rarely occur. Simple life forms might appear under a variety of circumstances, but complex life is extraordinarily atypical [51]. This would account for the apparent absence of evidence of extraterrestrial life anywhere in the solar system and beyond. But even Ward and Brownlee believe that microorganisms probably emerged in many environments beyond Earth; they have just not been discovered yet.

Some religious leaders also opposed the search for life elsewhere in the cosmos not for scientific reasons, but because they believed that the discovery of extraterrestrial life could be damaging for much of the religious community. While many religious might be open to this determination, some fundamentalists viewed extraterrestrial life as a undermining of the gospel, especially sacred texts which make no mention of it. It also tends to demote humanity from its privileged place in relation to God. In so doing, it could minimize humanity’s role in the cosmos andthe reason for human existence. It could even demote the place of Jesus Christ as Savior; finding extraterrestrials with technologies far beyond our own that might offer “technological salvation” for humankind. Such would be anathema in some religious settings [52]. 

This begs a fundamental question: how do religious individuals justify the search for life on planets such as Mars and perhaps Venus? Sometimes they accept the fact that life beyond Earth was created by God just as it was on Earth [53]. For instance, James C. Fletcher, NASA administrator between 1971 and 1976 and again in 1986-1989, was enthusiastic about this search for life beyond Earth and supported the Viking mission to Mars because of it. His Mormon faith explicitly asserted the existence of life on other planets. Established early on as a part of the Mormon faith, therefore, was the idea of a plurality of worlds inhabited by other beings [54]. 

Fletcher was committed to the ideas expressed in Mormon religion and emphasized the belief that humans were not alone as intelligent beings in the universe. He was interested in the probability of finding other civilizations in space and commented on it repeatedly. For example: “It is hard to imagine anything more important than making contact with another intelligent race. It could be the most significant achievement of this millennium, perhaps the key to our survival as a species” [55]. More recently Fletcher remarked that “intelligent life on other planets around other suns is a likely possibility” and he saw it as critical that NASA look for it. He added, “The public’s misconception is that they don’t realize how real a possibility such intelligence is” [56]. 

## 5. Conclusions

The search for life on Mars, and to a lesser extent on Venus, addresses seemingly deep-seated needs that strike at the confluence of the scientific pursuit of knowledge and the philosophical understanding of humanity’s place in the universe. According to planetary scientist Michael Caplinger:

The question of whether life is common or rare in the universe has deep philosophical implications. It is uncertain exactly how life arose on Earth, so it is difficult to determine how common such mechanisms are. But if life also arose on Mars, this would show that those mechanisms operated not just once, but twice, arguing that life may well be common elsewhere. However, the search for life on Mars thus far has been unsuccessful. Some portion of the scientific community feels that further searches are a waste of time, while another portion remains neutral or guardedly optimistic. In principle, it’s simple to prove that there *is* life on Mars—all one need do is find an example. Proving there isn’t life on Mars is much harder. Even a prolonged negative search can be countered with the suggestion of yet another, more inaccessible place in which to look. In the case of Mars, the issue has been complicated by the emotional belief in an Earthlike Mars, which has largely been shown to have been a myth. Mars is a spectacular place, and will remain so even if it is finally proved to be lifeless. Today, we don’t know for sure if there is or ever was life on Mars. But one thing is certain—one day, there will be [57].

This faith in positive results is repeated in many settings with similar expectations. Perhaps it is a part of the human condition. Perhaps it is somewhat like the tagline from the “X-Files,” the 1990s television series concerning the search for extraterrestrial visitation of Earth, “I Want to Believe.” We all collectively want to believe and are willingly expending millions of dollars each year seeking confirmation of that desire. Perhaps we will find someday, using another tagline from the “X-Files,” that, “The Truth is Out There.” Meantime, we investigate, and with every disconfirming piece of evidence modify the nature of the search slightly in cognitive dissonance as outlined in Leon Festinger’s work.
